# Cardioprotective Properties of *Ginkgo Biloba* Extract 80 *via* the Activation of AKT/GSK3β/β-Catenin Signaling Pathway

**DOI:** 10.3389/fmolb.2021.771208

**Published:** 2021-11-03

**Authors:** XiangWei Zheng, Qi Gao, Shuang Liang, GuoQin Zhu, DanDan Wang, Yi Feng

**Affiliations:** ^1^ Engineering Research Center of Modern Preparation Technology of Traditional Chinese Medicine, Ministry of Education, Innovation Research Institute of Traditional Chinese Medicine, Shanghai University of Traditional Chinese Medicine, Shanghai, China; ^2^ SPH Xing Ling Sci. and Tech, Pharmaceutical Co., Ltd., Shanghai, China

**Keywords:** Ginkgo biloba extract, myocardial protection, acute myocardial infarction, apoptosis, GSK3β, β-catenin, injection, signaling pathway

## Abstract

Elderly people are more likely to experience myocardial infarction (MI) than young people, with worse post-MI mortality and prognosis. *Ginkgo biloba* extract 50 (GBE50) is an oral GBE product that matches the German product, EGb761, which has been used to treat acute myocardial infarction (AMI). The extraction purity of GBE50 was improved to form a new formulation, *Ginkgo biloba* extract 80 (GBE80). This study investigates the effect of GBE80 on aged acute myocardial infarction rats. GBE80 injection is a novel formulation that was prepared by mixing Ginkgo flavonoids and lactones in a 4:1 weight ratio, with a Ginkgo content of more than 80%. Cell Counting Kit-8 was used to determine the biological safety and protective effect of GBE80 on cardiomyocytes against oxidative damage. An aged AMI rat model was developed and used to determine the myocardial infarction weight ratio using triphenyltetrazolium chloride staining. Terminal deoxynucleotidyl transferase-mediated dUTP-biotin nick end labeling (TUNEL) was applied to detect cell apoptosis in myocardial tissue. Western blotting and immunohistochemistry were used to measure the protein levels of members of the AKT/GSK3β/β-catenin pathway *in vitro* and *in vivo*, respectively. We found that GBE80 *in vitro* suppressed H_2_O_2_-induced cytotoxicity by promoting AKT/GSK3β/β-catenin signaling, while it did not show cytotoxicity to normal cardiomyocytes in the 0–500 μg/ml dose range. After 7 days of administration to aged AMI rats, GBE80 markedly reduced the weight ratio of the infarction and inhibited cell apoptosis in myocardial tissue. Furthermore, the AKT/GSK3β/β-catenin signaling pathway was activated by GBE80. These results suggest that GBE80 injection effectively inhibited AMI-induced myocardial damage and *in vitro* H_2_O_2_-induced cardiomyocyte cytotoxicity by activating the AKT/GSK3β/β-catenin signaling pathway.

## Introduction

Myocardial infarction (MI) is a common clinical adverse cardiovascular event, which can endanger the lives of patients. The Framingham Heart Study indicated that higher age is significantly associated with risk of myocardial infarction (Ngwa et al., 2021). Compared with middle-aged men (55–64 years), the MI incidence of elderly men (85–94 years) was more than two times higher, and increased by more than five times for women (women aged 55–64 years *vs* 85–94 years) (National Heart, Lung, and Blood Institute, 2006; Qipshidze Kelm et al., 2018). The incidence of MI among the elderly is not only significantly higher than that among young people, but the mortality rate after MI is significantly higher than that of young people. The GISSI-2 (Gruppo Italiano per lo Studio della Sopravvivenza nell’Infarto Miocardico-2) study showed that the risk of cardiac rupture increases significantly with age in patients receiving thrombolytic therapy for reperfusion after a first MI. The mortality rates of both in-hospital and post-discharge patients increases with age, with a 6% mortality rate increase per year of age increase (Maggioni et al., 1993). Elderly patients are more prone to cardiac arrest after MI, which induces MI complications, including papillary muscle rupture, left ventricle free wall rupture, and acquired ventricular septal defect (Ornato et al., 2001). The proportion of the global population aged over 60 will increase from 10.0% in 2000 to 21.8% in 2050 and 32.2% in 2,100 (Lutz et al., 2008). These predictions have prompted researchers to seek more effective treatments for MI.

*Ginkgo biloba* L. is well known as a living fossil tree because of its longevity. Over time, ginkgo must have acquired or developed resistance to various diseases to adapt to the environment. *G. biloba* leaves contain many phytochemicals, including flavonoids, terpenoids, alkylphenols, and carboxylic acids (van Beek and Montoro, 2009; van Beek, 2000). The terpene lactones consist of bilobalide. Ginkgolides A, B, C, and J are only found in *G. biloba* trees. The major ginkgo flavonoids are mono-, di-, and tri-glycosides (van Beek and Montoro, 2009). The chemical components of the ginkgo leaf show a variety of activities, such as antioxidation, elimination of oxygen free radicals, regulation of superoxide dismutase and catalases, and elimination of nitric oxide (NO), thus contributing to protection against cardiac damage, and potentially preventing myocardial infarction (Tsai et al., 2013). The ginkgo leaf extract has been developed clinically as an important medicinal herb (World Health organization, 1999). A standardized ginkgo extract first appeared in 1994 in Germany, and has been approved for the treatment of, for example, cerebral insufficiency (Blumenthal, 1998).

In China, *Ginkgo biloba* Extract 50 (GBE50) is a representative ginkgo extract that contains 24.1% ginkgo-flavone glycosides (including kaempferol, quercetin, and isorhamnetin derivatives) and 6.4% lactones (including ginkgolides A, B, C, and bilobalide). Some studies have shown that GBE50 may prevent MI (Liu et al., 2010; Bian et al., 2018) by attenuating the abnormal expression of the Na^+^—Ca^2+^ exchanger (NCX) (Liu et al., 2013). GBE50 is also used to treat MI in traditional Chinese medicine (Chinese Pharmacopoeia Commission, 2005). Currently, the purity of the flavonoids and diterpenes in GBE50 is relatively low, and GBE50 is often used as an oral preparation. Oral GBE50 is often inconvenient for use in patients with acute myocardial infarction (AMI); rather, injectable medications that work quickly are often required for AMI. Although injection of the crude extract of Ginkgo biloba has been used to treat AMI, its composition is complex, resulting in a poor safety profile and an unclear mechanism of action.

*Ginkgo biloba* has been shown to activate AKT signaling pathway. It has been also widely demonstrated that the activation of AKT triggers intracellular events, such as the phosphorylation of glycogen synthase kinase 3β (GSK3β), which confers protection against AMI damage. Mahesh Thirunavukkarasu et al. showed that AKT signaling pathway is reduced by myocardial ischemia-reperfusion injury (Thirunavukkarasu et al., 2015). Thus, it would be interesting to study the activation of AKT and phosphorylated GSK3β (*p*-GSK3β) by *Ginkgo Biloba* extract on aged AMI rats.

The present study aimed to increase the purity of the flavonoids and diterpenes in GBE50 to more than 80%, and prepare an injection to investigate its effect on the treatment of AMI. Ginkgo biloba extract 80 (GBE80) is a new ginkgo leaf extract that was prepared by mixing Ginkgo flavonoids and lactones with a Ginkgo content of more than 80% in a weight ratio of 4:1, which was consistent with the yield ratio of ginkgo flavonol glycosides and ginkgolides of GBE50. The GBE80 injection was studied for its protective effect on cardiac damage caused by AMI *in vitro* and *in vivo*. The molecular mechanism was also explored.

## Materials and Methods

### Chemical Reagents

The suppliers and the catalog numbers of the reagents are as follows. Dulbecco’s modified Eagle’s medium (DMEM) (Life Technologies, Carlsbad, CA, United States ; 22,400–089), fetal bovine serum (Life Technologies; 10,099), antibiotic-antimycotic (Life Technologies; 15,240–112), phosphate-buffered saline (PBS) (Life Technologies; 10010-049, pH7.4), trypsin-EDTA (Life Technologies; 25300-054, 0.05%), bovine serum albumin (Life Technologies; 15560012); D-Hanks solution (Beyotime, Jiangsu, China; C0218); type II collagenase (Sigma, St. Louis, MO, United States ; C6885); 5-bromo-2'-deoxyuridine (5-BrdU) (Sigma; B5002); Cell Counting Kit-8 (CCK-8; DOJINDO, Kumamoto, Japan; CK04), IRDye 680CW (Licor, Lincoln, NE, United States ), protein molecular weight markers (Beyotime; P0066), BCA protein concentration determination kit (Solarbio, Beijing, China; PC0020). Antibodies: anti-glycogen synthase kinase beta (GSK3β) (Cell Signaling Technology (CST), Danvers, MA, United States ; 12,456), anti-AKT serine/threonine kinase 1 (AKT1) (CST; 2,967), anti-AKT serine/threonine kinase 2 (AKT2) (CST; 2,962), anti-β-catenin (Abcam, Cambridge, MA, United States ; ab24925), and anti-glyceraldehyde-3-phosphate dehydrogenase (GAPDH) (Abbkine, Wuhan, China; Abp57259).

### Preparation of GBE80 Injection

The purity of ginkgo flavonol glycosides in GBE50 (24%) was increased to more than 80% by modified macroporous resin (LSA-12S) column chromatography, and the purity of ginkgo ginkgolides was improved from 6% to over 80% by normal phase silica gel column chromatography combined with recrystallization, according to previously published methods (Zheng et al., 2018a; Zheng et al., 2018b)_._ Then, ginkgo flavonol glycosides and ginkgolides with purity higher than 80% were mixed in a ratio of 4:1 (mass ratio), which was consistent with the yield ratio of ginkgo flavonol glycosides and ginkgolides in GBE50. GBE80 (2.5 mg/ml) was dissolved in 10% dimethyl sulfoxide (DMSO) + 30% polyethylene glycol (PEG) aqueous solution, and 1.6 ml was diluted in 50 ml water to obtain semi-finished GBE80 injection product. The product was preliminarily filtered by precision filtration, and was immediately canned and sealed after passing the qualified filtration test. The GBE80 injection was steam sterilized at 100°C, and leak detection was performed after sterilization.

### Culture of Rat Neonatal Cardiomyocytes

The isolation and primary culture of rat neonatal cardiomyocytes were performed in accordance with previously published methods (Bergmann et al., 2004; Duan et al., 2015). The cell suspension was collected and mixed into the DMEM containing 10% (V/V) fetal bovine serum (FBS), passed through a 200-µm mesh sieve, and centrifuged at 1,000 rpm for 10 min. The cell pellets were resuspended and cultured in DMEM containing 10% FBS for 2 h to allow fibroblasts to adhere preferentially. Non-adherent cells were transferred into a new flask and cultured in DMEM containing 10% FBS and 0.1 μM of 5-BrdU, which was used to inhibit the growth of fibroblasts. Cells were passaged when they reached 80–90% confluence and cultured in DMEM containing 10% FBS without 5-BrdU. Cardiomyocytes at passage 3 to 5 were used in this study.

### Cell Viability Assay

The cells were seeded into a 96-well plate at 2000 cells/well. In the safety assay, serial dosages of GBE80 were used to treat the cardiomyocytes. In the viability assay, serial dosages (10 μg/ml, 30 μg/ml, and 100 μg/ml) of GBE80, GBE50, or captopril were added into each well together with 0.03% H_2_O_2_. After 48 h, 10 µl of CCK-8 reagent was added into each well and incubated for 4 h. The absorption at 450 nm was measured using a microplate reader.

### Animals

Healthy aged male Sprague-Dawley rats (aged 22–24 months, weight: 330 ± 530 g) were purchased from B&K universal Group Co. Ltd. (Shanghai, China). All rats were housed at a controlled temperature (22 ± 2°C), relative humidity (55 ± 5%), and 12-h light/dark cycle, and were allowed food and water *ad libitum*. All animal procedures were performed following the “Guidelines for the Care and Use of Laboratory Animals” of Shanghai University of Traditional Chinese medicine.

### Establishment of the Acute Myocardial Infarction (AMI) Model in Aged Rats

Coronary artery ligation was conducted to establish the AMI rat model according to the procedures of Chen et al. (2019). The aged rats were anesthetized intramuscularly using 10:1 tiletamine/zolazepam and xylazine. After left-sided thoracotomy in the fourth rib interspace, the left anterior descending coronary artery was ligated using a 6–0 silk thread, which was confirmed by the color change of the myocardial tissue. The chest and skin were closed using 2–0 sutures. For the sham (control) operation, a similar procedure was performed, but without ligation.

### Design and Allocation

After ligation, the rats were treated daily for seven consecutive days, followed by euthanasia after another month of follow-up. Seventy rats were randomly divided into seven groups (*n* = 10 per group): The sham group 1) and model group 2) were intravenously injected with 10% DMSO +30% PEG +60% saline solution (0.9%); 100 mg/kg GBE80 3), 30 mg/kg GBE80 4), 10 mg/kg GBE80 5) and 30 mg/kg captopril 7) were injected intravenously for 7 days; 30 mg/kg GBE50 6) was administered intragastrically with GBE80 for 7 days.

### Western Blotting Assay

Radioimmunoprecipitation assay (RIPA) lysis buffer was used to extract total proteins from the cultured cardiomyocytes. A BCA protein concentration determination kit was used for protein quantification. Equivalent amounts of protein (20 μg) were separated using 10% SDS-PAGE gels and transferred electrically onto a polyvinylidene fluoride (PVDF) membrane. After blocking using 5% fat milk powder, the PVDF membrane was incubated overnight in the primary antibody solution (4°C) with the anti-GAPDH antibody as the internal protein control. Then, the membrane was incubated with IRDye-680CW-conjugated secondary antibody and developed using the enhanced chemiluminescent (ECL) reagent at room temperature. The optical density of the immunoreactive protein bands was quantified using ImageJ software (NIH, Bethesda, MD, United States ) and normalized to the signal of GAPDH.

### Myocardial Infarction Size Measurement Using Triphenyltetrazolium Chloride (TTC)

Measurement of the infarction size was performed according to the procedure by van Rooij et al. (2002) and Yang et al. (2018). The left ventricle was cut transversely into 2–3 mm slices starting from the apex. The slices were incubated with 1% TTC at 37°C in the dark and then fixed using 4% paraformaldehyde solution for 8 h. The TTC unstained (white) part was the infarcted heart region, which was regarded as the infarction area (INF), while the stained (red) part was the normal heart tissue. The myocardial infarct size was expressed as the weight ratio: The total weight of INF/the total weight of the left ventricle.

### Terminal Deoxynucleotidyl Transferase-Mediated dUTP-Biotin Nick End Labeling (TUNEL) Assays

The left ventricle was cut into slices of 5-µm thickness using a microtome (CM 1900; Leica, Wetzlar, Germany) and then fixed overnight in 4% polyoxymethylene solution. A commercial TUNEL kit (Beyotime) was used to detect the apoptotic cells in the frozen myocardium. The ratio of the number of TUNEL positive cells to the total number of cells was analyzed using ImageJ.

### Heart Histological Assays

The left ventricle was fixed with 10% formaldehyde for 3 h at 4°C, and then paraffin slices were prepared by making the tissue transparent, embedding, and slicing. The paraffin slices were treated with citrate buffer for 20 min at 98°C to repair the antigens. After 15 min of blocking using 10% goat serum, the slices were incubated with the primary antibody overnight at 4°C. After washing three times with PBS-Tween 20 (PBST), the myocardium was incubated with the biotin-labeled secondary antibody at 37°C for 30 min. Then, the positive cells in the myocardium were stained using with 3,3′-diaminobenzidine (DAB) solution (Beijing Zhongshan Jinqiao Biotechnology Co., Ltd., Beijing, China). The quantitative analysis for positively stained cells was performed by Image-Pro Plus software (v6.0) to express results as average optical density (AOD) at a magnification of ×100.

### Statistical Analysis

Continuous variables were expressed as the mean ± standard deviation (SD). GraphPad Prism software 5.0 (GraphPad Inc., La Jolla, CA, United States ) was used to analyze the data using one-way analysis of variance (ANOVA). A *p* value ≤0.05 indicated that the difference was statistically significant.

## Results

### GBE80 Reduces Oxidation-Induced Injury in Cardiomyocytes

**FIGURE 1:**
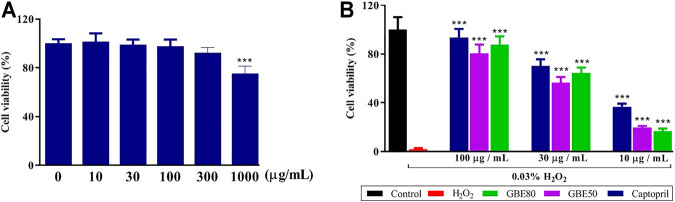
GBE80 treatment reduces oxidation-induced injury in cardiomyocytes. Cardiomyocyte viability and protective effect following exposure of the cells to GBE80 alone **(A)** or different treatments under H_2_O_2_ injury **(B)**, respectively. (*** indicates *p* < 0.001 *vs* the control group **(A)** or H_2_O_2_ group **(B)**, respectively).

When the concentration of GBE80 was lower than 1,000 μg/ml, the viability of cardiomyocytes was not affected, while GBE80 at 1,000 μg/ml decreased the cardiomyocyte viability to less than 80% (48 h, *p* < 0.05, Figure 1A). Therefore, in this study, 0–100 μg/ml GBE80 was used as the safety concentration. H_2_O_2_ at 0.03% induced significant oxidative damage in cardiomyocytes. GBE80, GBE50, and captopril reversed the cytotoxicity induced by H_2_O_2_, and at the same concentration, GBE80 had a better effect on cardiomyocyte viability than GBE50 (Figure 1B).

### GBE80 Treatment Activates AKT/GSK3β/β-Catenin Signaling in Cardiomyocytes

**FIGURE 2:**
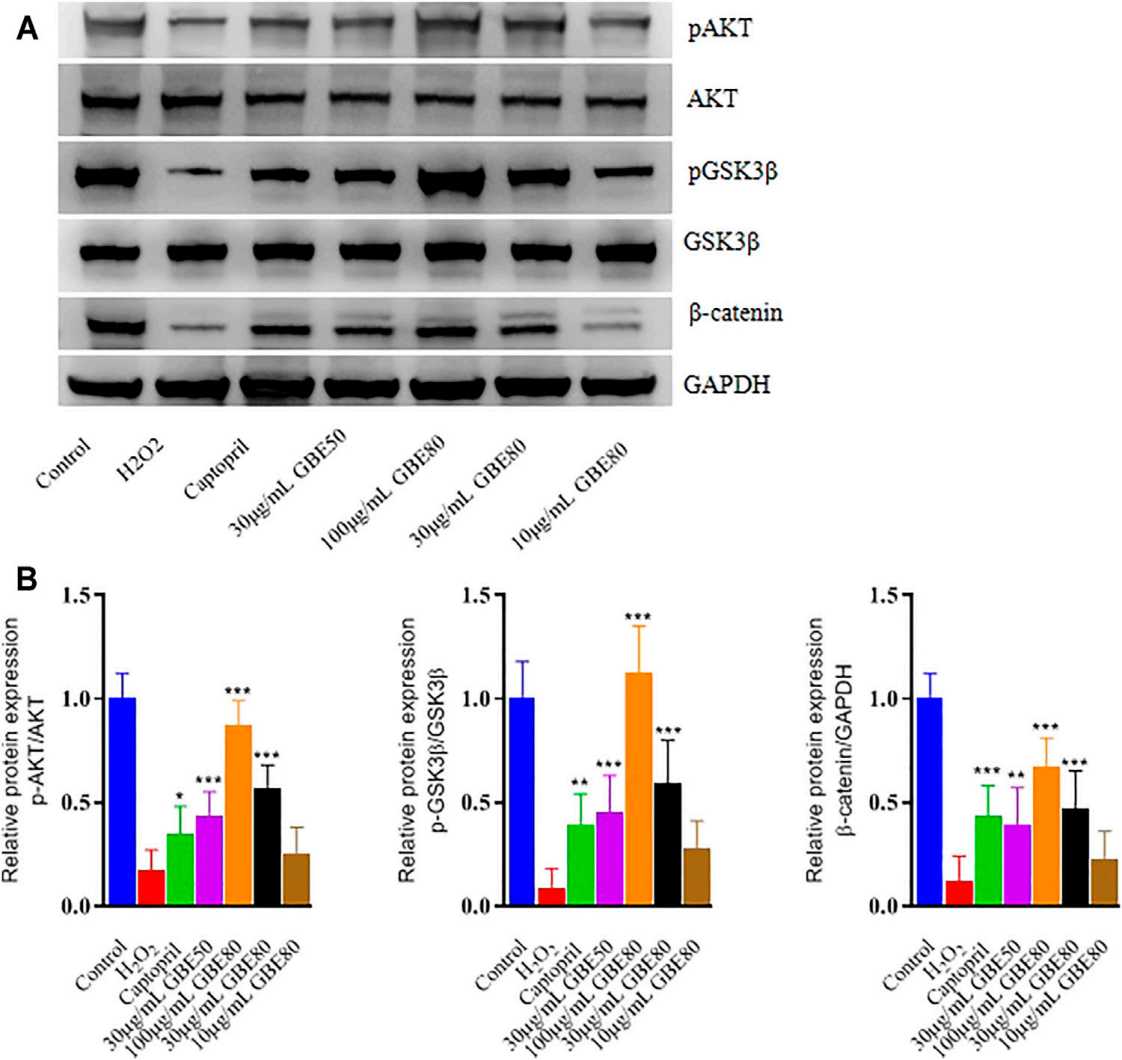
Effects of different treatments under H_2_O_2_ injury on the levels of pAKT, AKT, pGSK3β, GSK3β, and β-catenin. Western blotting **(A)** and densitometry analysis **(B)** of pAKT, pGSK3β, and GAPDH in cardiomyocytes treated with H_2_O_2_ and different treatment. (*, **, *** indicates *p* < 0.05, *p* < 0.01, *p* < 0.001 *vs* the H_2_O_2_ group, respectively.)

The levels of phosphorylated (*p*) AKT, pGSK3β, and β-catenin decreased when the cardiomyocytes were treated with H_2_O_2_. Western blotting confirmed that GBE80 restored the decreased levels of pAKT, pGSK3β, and β-catenin caused by oxidative damage (Figure 2). In addition, GBE80 significantly increased pAKT, pGSK3β, and β-catenin proteins levels in a dose-dependent manner. These results revealed that GBE80 activated the AKT/GSK3β/β-catenin signaling pathway, and played an antioxidant role in cardiomyocytes *in vitro*.

### GBE80 Administration Exerts Beneficial Effects on the Myocardial Infarction Size

**FIGURE 3:**
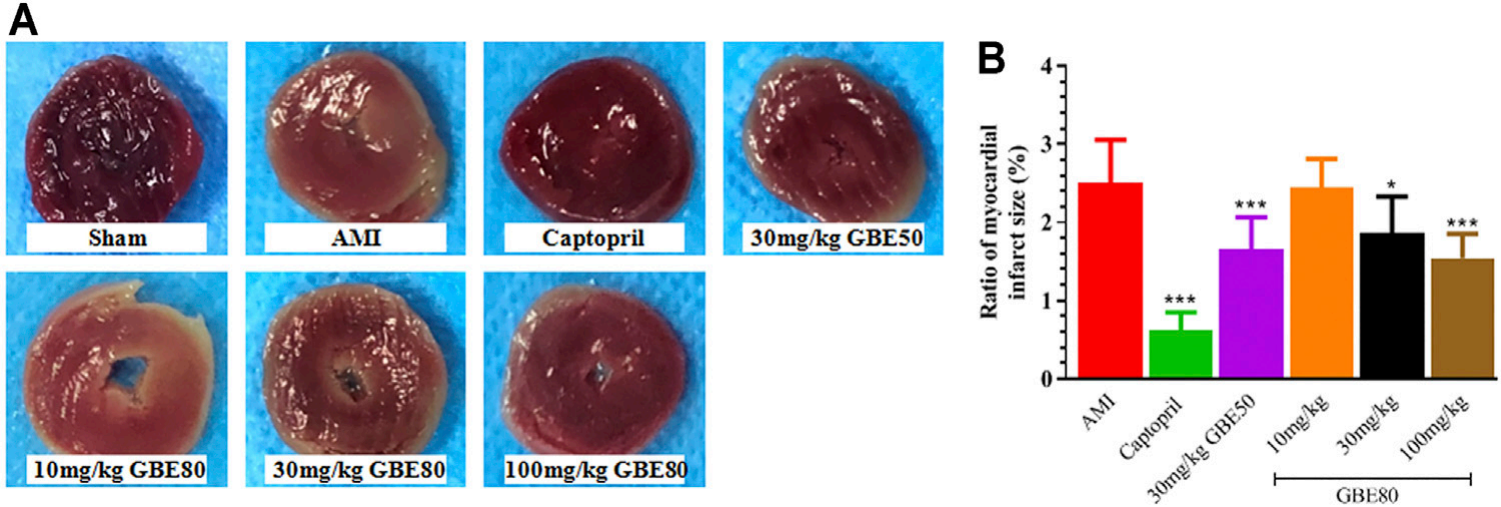
GBE80 administration exerts beneficial effects on the size of the myocardial infarction. **(A)** Representative figures of the triphenyltetrazolium chloride (TTC)-stained myocardial tissues of acute myocardial infarction (AMI) rats receiving indicated treatment; and quantitative **(B)** statistical analysis of the myocardial infarct size. (*, *** indicates *p* < 0.05, *p* < 0.001 *vs* the AMI group, respectively).

The TTC staining results shown in Figure 3A were representative slices that delineated the heart infarction size. Obviously, the myocardial slice of the sham group did not present a pale infarct area, whereas the pale infarction area was distinct in the model group slice. The middle and high doses of GBE80 effectively reduced the area of the myocardial infarction dose-dependently. Quantitative analysis further confirmed that the weight ratio of the myocardial infarction in the GBE80 treatment group was significantly smaller than that of the model group (*p* < 0.05, Figure 3B), which indicated that GBE80 could protect the myocardial tissue of the AMI model rats.

### TUNEL Staining Shows That GBE80 Treatment Reduces Myocardial Injury After AMI

**FIGURE 4:**
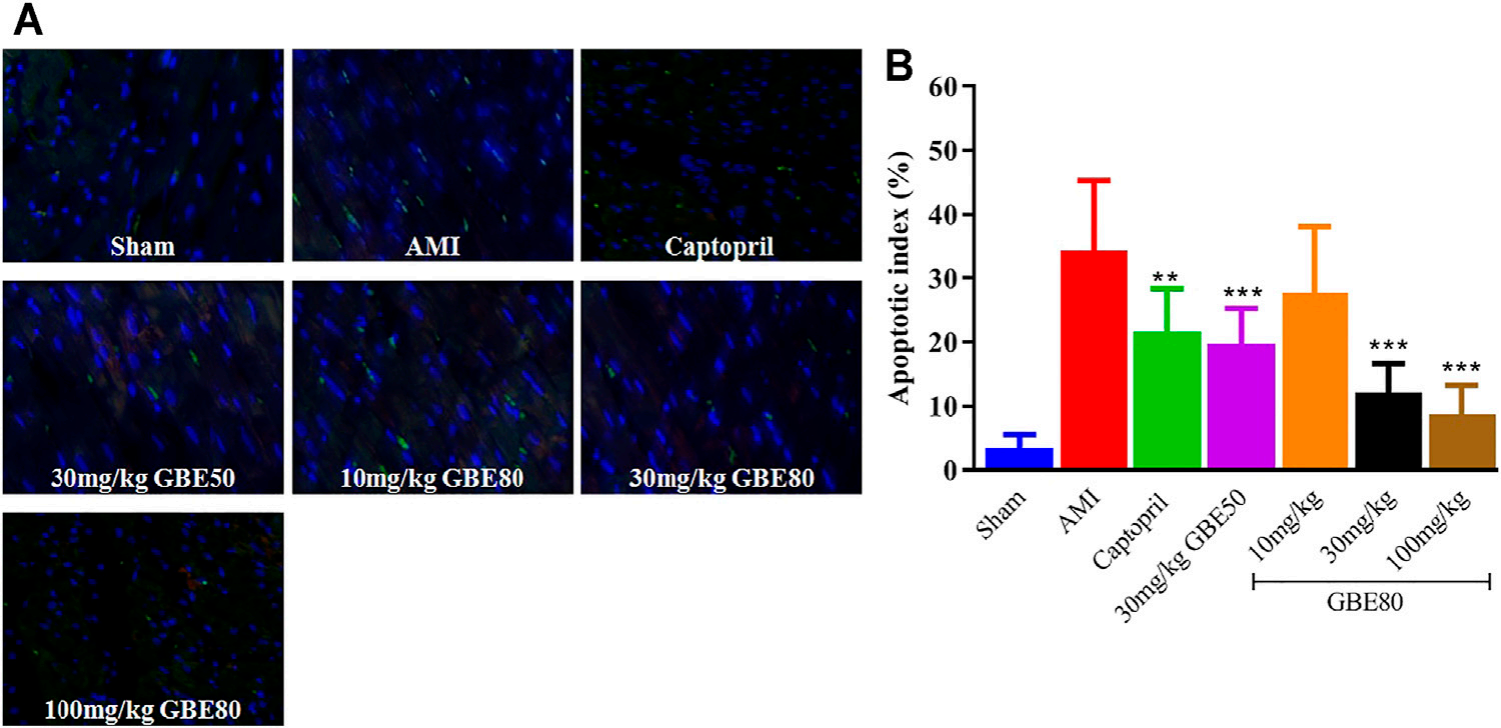
GBE80 inhibits cardiomyocyte apoptosis of the left ventricle in AMI. **(A)** Representative figures of TUNEL-stained myocardial tissues of acute myocardial infarction (AMI) rats receiving the indicated treatment (×200). **(B)** Quantitative statistical analysis of cardiomyocyte apoptosis. (**, *** indicates *p* < 0.01, *p* < 0.001 *vs* the AMI group, respectively).

The TUNEL staining results showed that AMI caused cardiomyocyte apoptosis, and the TUNEL-positive nuclei ratio in myocardial tissue was suppressed significantly after intravenous injection of GBE80 for 7 days (Figure 4A). With the increase in GBE80 dose, the ratio of TUNEL-positive nuclei decreased significantly (Figure 4B), which indicated that GBE80 treatment had a protective effect on the myocardial tissue of aged rats.

### *In vivo* GBE80-Related Effects on AKT/GSK3β/β-Catenin Signaling Activity

**FIGURE 5:**
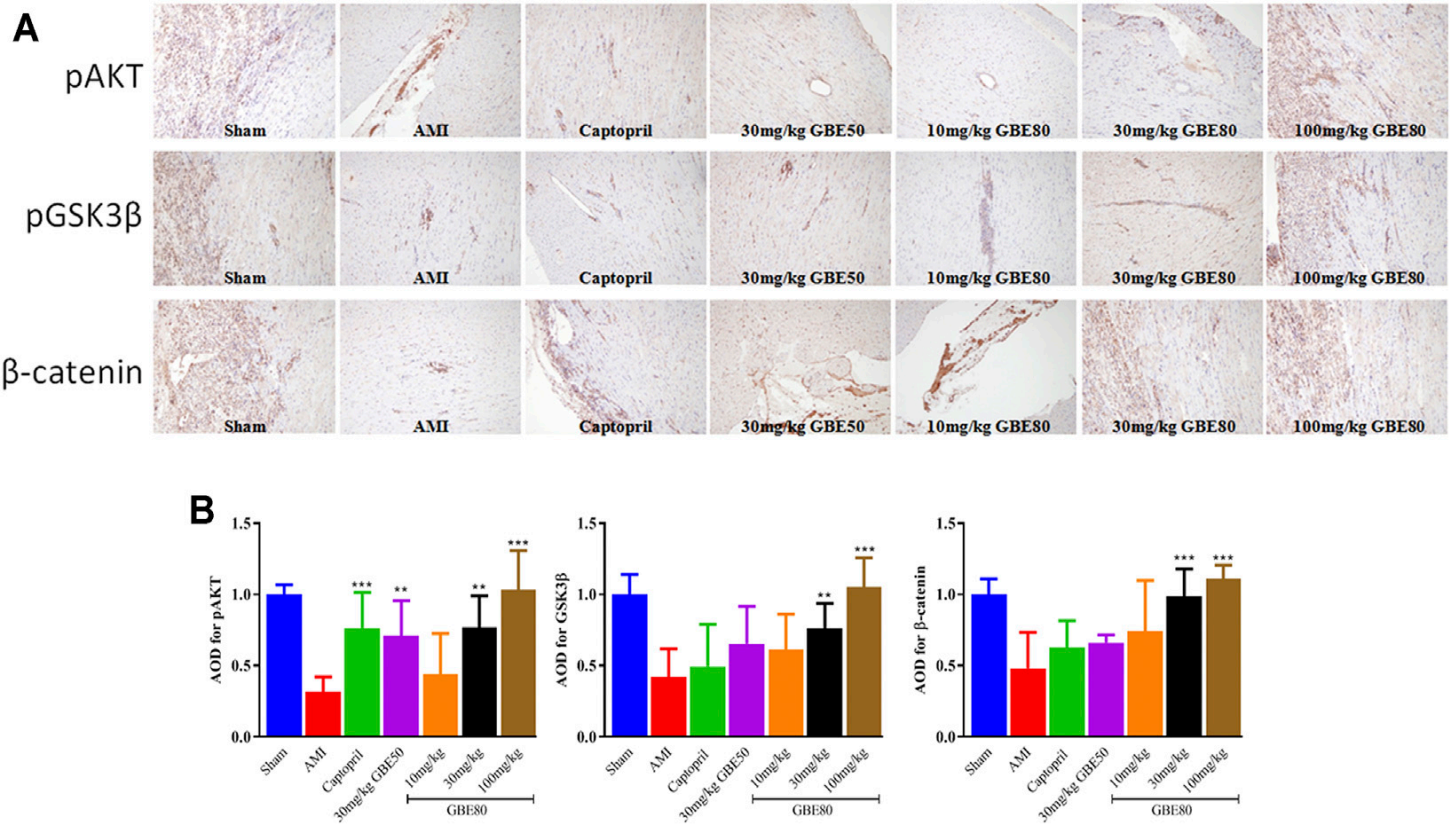
Expressions of pAKT, pGSK3β and β-catenin in the left ventricle detected by immunohistochemical staining. **(A)** Representative immunohistochemical staining obtained from left ventricles of several groups with pAKT, pGSK3β and β-catenin detection (×100). **(B)** Quantitative statistical analysis of AOD values using Image-Pro Plus software. (**, *** indicates *p* < 0.01, *p* < 0.001 *vs* the AMI group, respectively).

The molecular mechanism of GBE80 myocardial protection was studied by detecting the levels of AKT/GSK3β/β-catenin pathway-related proteins. The levels of GSK3β, AKT, and β-catenin in the model group were reduced significantly compared with those in the sham group, which confirmed that AKT/GSK3β/β-catenin signaling was affected by AMI injury (Figure 5A). GBE80 treatment resulted in a dose-dependent increase of GSK3β, AKT, and β-catenin levels in aged AMI rats. These results suggested that GBE80 might protect myocardial tissue in AMI by activating the AKT/GSK3β/β-catenin pathway.

## Discussion

The present study aimed to investigate the protective effects of GBE80 injection on acute myocardial ischemia injury and its possible regulatory mechanisms *in vivo* and *in vitro*. GBE80 showed a direct protective effect on H_2_O_2_-induced cardiomyocyte injury *in vitro*, as well as activated the AKT/GSK3β/β-catenin signaling pathway in cardiomyocytes. In the *in vivo* aged AMI model, GBE80 effectively reduced the infarction size and cell apoptosis in myocardial tissues, thereby protecting myocardial tissue. The protective mechanism of GBE80 might be related to the activation of the AKT/GSK3β/β-catenin signaling pathway, thus accelerating the recovery of myocardial tissue. In addition, GBE80 had no cytotoxicity toward cardiomyocytes in the 0–500 μg/ml dose range.

AMI is associated with contractile dysfunction and myocardial death, and therefore, post-ischemic myocardial recovery is of paramount importance (Symons et al., 2018). Interestingly, Liu et al. showed AMI with GBE50 treatment resulted in a significantly lower left ventricular systolic pressure (LVSP) compared with the baseline in the vehicle-treated group, and might exert its beneficial effects by acting as a negative inotropic agent and reducing oxygen consumption during ischemia (Liu et al., 2013). This is consistent with a previous report which showed that GBE50 prominently inhibited the contractive force of the right atrium in a concentration-dependent manner (Wang et al., 2010). In this study, we aimed to refine the purification of GBE50 to form GBE80. The purity of flavonoids and diterpenes contained in GBE80 was greater than 80%, and their mass ratio was equivalent to that of GBE50. The results showed that injection of GBE80 had a protective effect against myocardial tissue damage caused by AMI in aged rats, demonstrated by a reduction in infarct size and decreased cell apoptosis in myocardial tissue. This suggested that the protective effect of GBE80 might result from the same compound composition as GBE50.

AKT is closely related to cell proliferation, and plays an important role in promoting cell growth, inhibiting cell apoptosis, and maintaining cell survival (van Rooij, et al., 2002; World Health organization, 1999). A variety of *in vivo* and *in vitro* studies have confirmed that drug therapy and ischemic preconditioning can improve cardiac systolic and diastolic function, and inhibit cell apoptosis by activating AKT-related signaling pathways (Blumenthal, 1998; Osaki et al., 2004). GSK-3β increases the permeability of the mitochondrial membrane and promotes cell apoptosis (Duan, et al., 2015; Scarabelli et al., 2004). However, AKT phosphorylates GSK3β, which can inactivate and inhibit GSK3β to block apoptosis (McMurray and Swedberg, 2010). In addition, GSK3β phosphorylation results in the dissociation of β-catenin from the GSK3β complex and its transfer into the nucleus (vanBeek, 2000). The upregulated β-catenin in the nucleus activates T-cell factor (TCF) transcription factors, which are responsible for activating endothelial nitrous oxide synthase (eNOS), BCL2 apoptosis regulator (BCL2), and other heart-protective proteins. In the present study, GBE80 activated the AKT/GSKβ/β-catenin pathway, and we speculated that the activation of this pathway was associated with the protective efficacy of GBE80 against AMI.

Previous studies have shown that oral GBE50 is metabolized to flavonol aglycone conjugates in the liver, which play an important pharmacological role in the plasma and are believed to be important substances for the cardiac protection offered by GBE50. However, in the present study, GBE80 was administered by injection, resulting in the plasma mainly containing prototype flavonoids and diterpenes (Li et al., 2012; Chen et al., 2013). It was shown that even at the same dose (30 mg/kg), the higher concentrations of flavonoids and diterpenes were also responsible for the protective effect on the heart, because the compounds are purer in GBE80 than in GBE50.

GBE50, as an oral preparation, is inconvenient for patients with severe disease, such as AMI. Although injection of the crude extract from *G. biloba* leaves has been used to treat AMI, its complex composition, poor safety, and unclear mechanism of action have limited its application. In the present study, the GBE50 oral formulation was changed to an injectable formulation, which could be applied to patients who may not be suitable for oral administration, could rapidly achieve efficacy in the body, and could be used to determine the accurate composition of the substances that exert a protective role in plasma. In addition, because of the relatively lower purity of flavonoids and diterpenoids in GBE50, the purities of these two compounds were increased to more than 80%, which laid the foundation for drug delivery at higher concentrations and improved the extraction technique from *G. biloba* leaves.

In summary, GBE80 inhibits the apoptosis of myocardial cells by activating the AKT/GSK3β/β-catenin pathway, thereby protecting cardiomyocytes from cardiac injury caused by AMI. Furthermore, the injectable form of GBE80 provides another option for the clinical application of *G. biloba* extract to treat AMI.

## Data Availability

The original contributions presented in the study are included in the article/Supplementary Material, further inquiries can be directed to the corresponding authors.

## References

[B1] Bergmann M. W. Rechner C. Freund C. Baurand A. El Jamali A. Dietz R. (2004). Statins Inhibit Reoxygenation-Induced Cardiomyocyte Apoptosis: Role for Glycogen Synthase Kinase 3β and Transcription Factor β-Catenin. J. Mol. Cell Cardiol. 37, 681–690. 10.1016/j.yjmcc.2004.05.025 15350841

[B2] Bian J. Zhao L. N. Yuan F. Y. Wem L. Q. Chen Y. (2018). Protective Effect of Pretreatment with Ginkgo Biloba Extract on Myocardial Tissue of Rats with Myocardial Ischemia Reperfusion and its Possible Mechanism. Prog. Anatomical Sci. 2406, 581–583+587. 10.16695/j.cnki.1006-2947.2018.06.006

[B3] Chen F. Li L. Xu F. Sun Y. Du F. Ma X. (2013). Systemic and Cerebral Exposure to and Pharmacokinetics of Flavonols and Terpene Lactones after Dosing Standardized G Inkgo Biloba Leaf Extracts to Rats via Different Routes of Administration. Br. J. Pharmacol. 170, 440–457. 10.1111/bph.12285 23808355 PMC3834766

[B4] Chen G. Liu G. Cao D. Jin M. Guo D. Yuan X. (2019). Polydatin Protects against Acute Myocardial Infarction-Induced Cardiac Damage by Activation of Nrf2/HO-1 Signaling. J. Nat. Med. 73, 85–92. 10.1007/s11418-018-1241-7 30191382

[B5] Chinese Pharmacopoeia Commission (2005). Pharmacopoeia of the People’s Republic of China. English Edition 2005, I. Beijing: People’s Medical Publishing House, 322–323.

[B6] Duan Z.-Z. Li Y.-H. Li Y.-Y. Fan G.-W. Chang Y.-X. Yu B. (2015). Danhong Injection Protects Cardiomyocytes against Hypoxia/Reoxygenation- and H2O2-Induced Injury by Inhibiting Mitochondrial Permeability Transition Pore Opening. J. Ethnopharmacology 175, 617–625. 10.1016/j.jep.2015.08.033 26320687

[B7] Li L. Zhao Y. Du F. Yang J. Xu F. Niu W. (2012). Intestinal Absorption and Presystemic Elimination of Various Chemical Constituents Present in GBE50 Extract, a Standardized Extract of Ginkgo Biloba Leaves. Curr. Drug Metab. 13, 494–509. 10.2174/1389200211209050494 22292790

[B8] Liu A.-H. Bao Y.-M. Wang X.-Y. Zhang Z.-X. (2013). Cardio-Protection by Ginkgo Biloba Extract 50 in Rats with Acute Myocardial Infarction Is Related to Na+-Ca2+ Exchanger. Am. J. Chin. Med. 41, 789–800. 10.1142/s0192415x13500535 23895152

[B9] Liu A. H. Zhang Z. X. Wang X. Y. (2010). Effect of GBE50 on Delayed Rectifier Potassium Current of Ventricular Myocytes in Ischemic guinea Pig. Zhongguo Ying Yong Sheng Li Xue Za Zhi 26, 444–448. 10.13459/j.cnki.cjap.2010.04.006 21328983

[B10] Lutz W. Sanderson W. Scherbov S. (2008). The Coming Acceleration of Global Population Ageing. Nature 451, 716–719. 10.1038/nature06516 18204438

[B11] Blumenthal M. (Editor) (1998). “The Complete German Commission E,” Monographs:Therapeutic Guide to Herbal Medicines (New York: American Botanical Council and Integrative Medicine CommunicationsAustin and Boston), 136–138.

[B12] Maggioni A. A. Maseri A. Fresco C. Franzosi M. G. Mauri F. Santoro E. (1993). Age-Related Increase in Mortality Among Patients with First Myocardial Infarctions Treated with Thrombolysis. N. Engl. J. Med. 329, 1442–1448. 10.1056/nejm199311113292002 8413454

[B13] McMurray J. J. V. Swedberg K. (2010). HEAAL: The Final Chapter in the Story of Angiotensin Receptor Blockers in Heart Failure-Lessons Learnt from a Decade of Trials. Eur. J. Heart Fail. 12, 99–103. 10.1093/eurjhf/hfp197 20083618

[B14] National Heart, Lung, and Blood Institute (2006). Incidence and Prevalence: 2006 Chart Book on Cardiovascular and Lung Diseases. Bethesda, MD: National Institutes of Health.

[B15] Ngwa J. S. Cabral H. J. Cheng D. M. Gagnon D. R. LaValley M. P. Cupples L. A. (2021). Revisiting Methods for Modeling Longitudinal and Survival Data: Framingham Heart Study. BMC Med. Res. Methodol. 21, 29. 10.1186/s12874-021-01207-y 33568059 PMC7876802

[B16] Ornato J. P. Peberdy M. A. Tadler S. C. Strobos N. C. (2001). Factors Associated with the Occurrence of Cardiac Arrest during Hospitalization for Acute Myocardial Infarction in the Second National Registry of Myocardial Infarction in the US. Resuscitation 48, 117–123. 10.1016/s0300-9572(00)00255-0 11426473

[B17] Osaki M. Oshimura M. Ito H. (2004). PI3K-Akt Pathway: Its Functions and Alterations in Human Cancer. Apoptosis 9, 667–676. 10.1023/b:appt.0000045801.15585.dd 15505410

[B18] Qipshidze Kelm N. Piell K. M. Wang E. Cole M. P. (2018). MicroRNAs as Predictive Biomarkers for Myocardial Injury in Aged Mice Following Myocardial Infarction. J. Cel Physiol 233, 5214–5221. 10.1002/jcp.26283 29150941

[B19] Scarabelli T. M. Stephanou A. Pasini E. Gitti G. Townsend P. Lawrence K. (2004). Minocycline Inhibits Caspase Activation and Reactivation, Increases the Ratio of XIAP to smac/DIABLO, and Reduces the Mitochondrial Leakage of Cytochrome C and Smac/DIABLO. J. Am. Coll. Cardiol. 43, 865–874. 10.1016/j.jacc.2003.09.050 14998631

[B20] Symons R. Claus P. Marchi A. Dresselaers T. Bogaert J. (2018). Quantitative and Qualitative Assessment of Acute Myocardial Injury by CMR at Multiple Time Points after Acute Myocardial Infarction. Int. J. Cardiol. 259, 43–46. 10.1016/j.ijcard.2018.02.093 29506936

[B21] vanBeek T. A. (Editor) (2000). Ginkgo Biloba (Amsterdam, Netherlands: Harwood Academic Publishers), 132–133.

[B22] Thirunavukkarasu M. Selvaraju V. Tapias L. Sanchez J. A. Palesty J. A. Maulik N. (2015). Protective Effects of Phyllanthus Emblica against Myocardial Ischemia-Reperfusion Injury: the Role of PI3-Kinase/Glycogen Synthase Kinase 3β/β-Catenin Pathway. J. Physiol. Biochem. 71 (4), 623–633. 10.1007/s13105-015-0426-8 26342597

[B23] Tsai H.-Y. Huang P.-H. Lin F.-Y. Chen J.-S. Lin S.-J. Chen J.-W. (2013). Ginkgo Biloba Extract Reduces High-Glucose-Induced Endothelial Reactive Oxygen Species Generation and Cell Adhesion Molecule Expression by Enhancing HO-1 Expression via Akt/eNOS and P38 MAP Kinase Pathways. Eur. J. Pharm. Sci. 48, 803–811. 10.1016/j.ejps.2013.01.002 23357604

[B24] van Beek T. A. Montoro P. (2009). Chemical Analysis and Quality Control of Ginkgo Biloba Leaves, Extracts, and Phytopharmaceuticals. J. Chromatogr. A 1216, 2002–2032. 10.1016/j.chroma.2009.01.013 19195661

[B25] van Rooij E. Doevendans P. A. de Theije C. C. Babiker F. A. Molkentin J. D. de Windt L. J. (2002). Requirement of Nuclear Factor of Activated T-Cells in Calcineurin-Mediated Cardiomyocyte Hypertrophy. J. Biol. Chem. 277, 48617–48626. 10.1074/jbc.m206532200 12226086

[B26] Wang X. Zhang Z. Liu A. (2010). Effects of GBE50 on Physiological Characteristics and Intracellular Free Calcium of Myocardium. Zhongguo Zhong Yao Za Zhi 35, 1866–1870. 10.4268/cjcmm20101422 20939287

[B27] World Health organization (1999). WHO Monographs on Selected Medicinal Plants, 1, 154–167. Geneva: World Health Organization

[B28] Yang G. Min D. Yan J. Yang M. Lin G. (2018). Protective Role and Mechanism of Snakegourd Peel against Myocardial Infarction in Rats. Phytomedicine 42, 18–24. 10.1016/j.phymed.2018.03.014 29655684

[B29] Zheng X. W. Gao Q. Zhu G. Q. Wei Y. F. Feng Y. (2018a). Preparation of High Purity Ginkgo Flavonol Glycosides by Acid/Polar Coupled Modified Macroporous Resin Column Chromatography. Chin. J. Pharmaceuticals 4909, 1283–1288. 10.16522/j.cnki.cjph.2018.09.012

[B30] Zheng X. W. Wu P. Y. Wang J. Wang D. D. Ruan K. F. Liang S. (2018b). Study on Preparation of High Purity Ginkgolide by Atmospheric Pressure Silica Gel Column Chromatography. China Med. Herald 1531, 30–33. CNKI:SUN:YYCY.0.2018-31-008.

